# The Odf2 status contributes to asymmetric inheritance of CD133-positive recycling endosomes during cytokinesis in SK-N-DZ human neuroblastoma cells

**DOI:** 10.1016/j.isci.2026.117062

**Published:** 2026-08-06

**Authors:** Hideki Izumi, Takao Mae, Sachiko Tsukita, Akira Nakagawara

**Affiliations:** 1 Laboratory of Molecular Medicine, Medical Research Institute, Saga Medical Center KOSEIKAN, Saga, Japan; 2 Saga Medical Center KOSEIKAN, Saga, Japan; 3 Advanced Comprehensive Research Organization, Teikyo University, Tokyo, Japan; 4 Graduate School of Frontier Biosciences, Osaka University, Osaka, Japan; 5 Saga HIMAT, Tosu, Japan

**Keywords:** centrosome, Odf2, CD133, recycling endosome, asymmetric cell division, neuroblastoma

## Abstract

Asymmetric cell division (ACD) is a physiological event that maintains development and tissue homeostasis. ACD generates two unequal daughter cells, one of which has stem cell properties while the other has differentiation potential. Recent studies show that the balance between self-renewal and differentiation is precisely controlled and that the disruption of this balance leads to diseases, such as tumors. Herein, we examined centrosome inheritance during cell division by using the human neuroblastoma cell line SK-N-DZ. The results showed that daughter cells inheriting daughter centrosomes with younger mother centrioles received more CD133-recycling endosomes, while those inheriting mother centrosomes with older mother centrioles received fewer CD133-recycling endosomes. The depletion of outer dense fiber protein 2/cenexin, which localizes to both centrosomal distal and subdistal appendages, strongly promoted the localization of recycling endosomes at both centrosomes, and ACD was lost. Therefore, these results may facilitate further research on the mechanisms underlying centrosome inheritance in ACD.

## Highlights

• Centrosomal appendages show asymmetric features during cytokinesis in SK-N-DZ cells
• CD133-recycling endosomes localize to centrosomes with immature centrosome appendages
• Odf2 depletion causes accumulation of CD133/Rab11-recycling endosomes at centrosomes
• Odf2 knockdown suppresses the asymmetric CD133 inheritance, promoting SCD

## Graphical abstract

**Figure:**
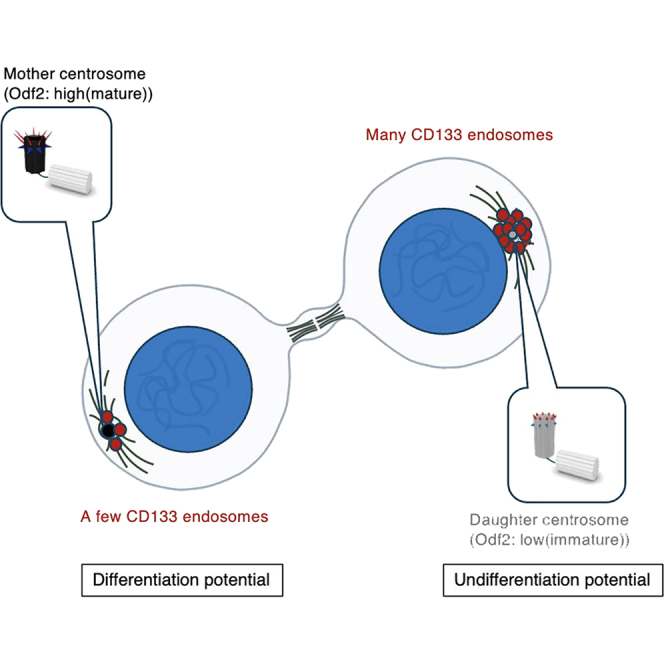

## Teaser

Biological sciences; Cell biology; Stem cells research

## Introduction

Asymmetric cell division (ACD) represents a pivotal process that is fundamental to development and tissue homeostasis across a wide range of organisms.^1^^,^^2^ ACD is a strategy that maintains the correct number of self-renewing stem cells and differentiated cells in a single division.^1^^,^^2^ Therefore, ACD balances the stem cell pool with its progenitor pool. However, the dysregulation of this balance has been implicated in developmental disorders, including tumorigenesis and/or cancer progression.^3^^,^^4^^,^^5^ In addition, ACD has been suggested to cause tumor cell heterogeneity in cancer cell populations.^6^

Neuroblastoma (NB), the most common solid tumor in children,^7^^,^^8^ provides an intriguing model to study ACD in the context of cancer.^9^^,^^10^^,^^11^ NB is derived from the multipotent neural crest cells that make up the structure of the embryo.^12^ The neural crest is composed of a cell population that produces multicell lineages. Therefore, NB cells are suspected to be similar in nature to cancer stem cells because of their pluripotent function.^12^ In addition, NB cell lines have unique properties because they exhibit immortal cell proliferation and are induced to become mature neurons by drugs such as retinoic acids.^12^ In human NB cells, *MYCN* expression regulates cell division fate.^9^^,^^13^ The overexpression of *MYCN* induces symmetric cell division (SCD) (self-renewal division), and the down-regulated expression of *MYCN* causes ACD.^9^ Furthermore, the transcriptional activity of MYCN is important for inducing SCD in human NB cells.^9^ The SK-N-DZ NB cell line, derived from NB, has been valuable for elucidating the dynamics of cell division and the interplay between self-renewal and differentiation.^10^ Despite advances in our understanding of the mechanisms governing ACD, the intricate processes responsible for organelle inheritance during cell division remain unclear, particularly the inheritance of centrosomes^14^ and associated organelles such as endosomes.^15^^,^^16^

**Figure 1:**
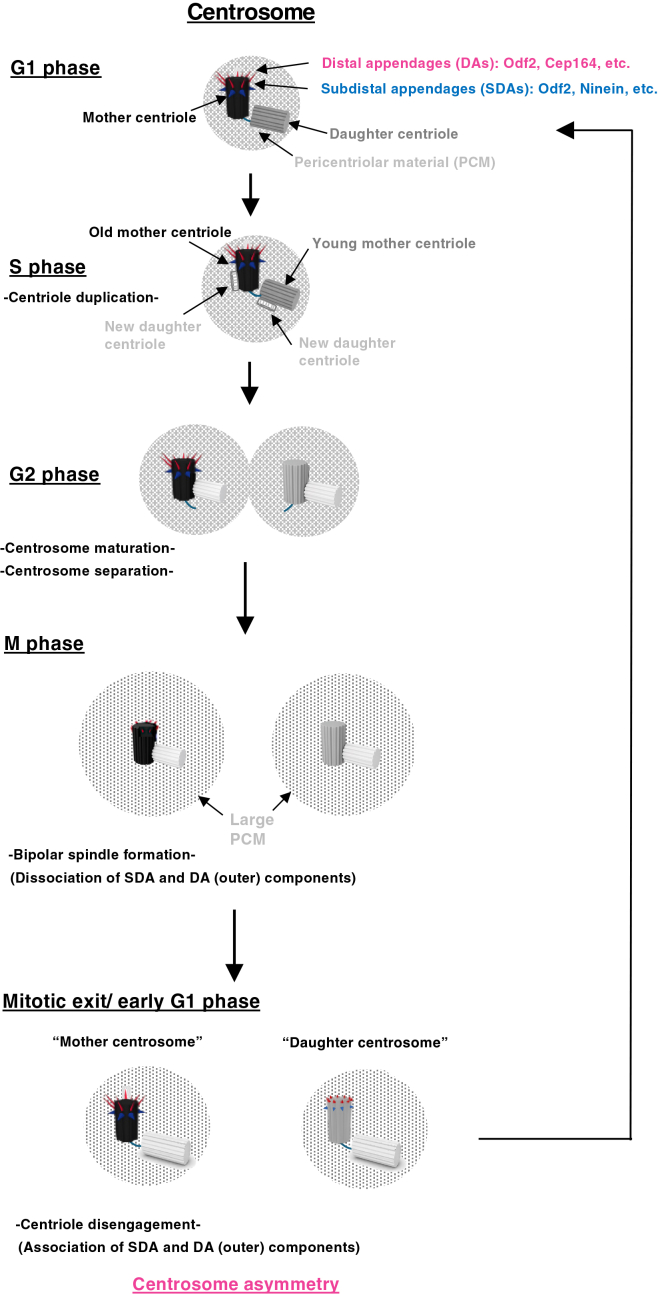
The centrosome status during the cell cycle The centrosome is composed of two centrioles and the pericentriolar material (PCM). The mother centriole has distal and subdistal appendages (DAs and SDAs), while the daughter does not. Centrosome duplication is initiated at the G1/S transition and occurs during the S phase, new daughter centrioles begin to be constructed, and the PCM of duplicated centrosomes mature during the G2 phase in the cell cycle. Duplicated centrosomes subsequently form bipolar spindles during the M phase (mitosis). The composition and structure of DAs and SDAs change throughout the cell cycle.^19^ DA outer components, such as Cep164, lose their DA localization in the G2 phase and re-associate on mature centrioles at mitotic exit.^20^^,^^21^^,^^22^ On the other hand, DA inner proteins appear to be permanently recruited to the mature centriole, maintaining the 9-fold structure of the DA during the cell cycle.^20^^,^^21^ The SDA structure also disappears in the G2 phase and re-appears during mitotic exit.^23^^,^^24^ Importantly, ODF2 remains localized to the centrioles during the cell cycle.^20^^,^^21^^,^^25^ The construction of DAs and SDAs on the young mother centriole occurs slowly during the M phase. Therefore, the distinction between old mother and young mother centrioles may be defined by the immunostaining signals of the DA and SDA proteins during cytokinesis. This distinction shows centrosome asymmetry.

The centrosome, a major microtubule-organizing center, plays a critical role in the spatial orientation and temporal regulation of cell division. The centrosome consists of mother and daughter centrioles as well as pericentriolar material (PCM)^17^^,^^18^ (Figure 1). The mother centriole has distal appendages (DAs) and subdistal appendages (SDAs) at its distal ends. DAs are required for basal body docking during ciliogenesis^26^ and SDAs are involved in the anchoring of microtubules to the centrosome.^27^ Centrosome ages differ between a centrosome with an old mother centriole (mother centrosome) and a centrosome with a young mother centriole (daughter centrosome).^20^^,^^28^^,^^29^ As shown in Figure 1, centrosome duplication is initiated at the G1/S transition and occurs during S phase; new daughter centrioles begin to be constructed, and the PCM of duplicated centrosomes matures during the G2 phase of the cell cycle.^30^ However, young mother centriole maturation occurs slowly during mitosis; when DAs and SDAs are matured in the mother centrioles, the construction of DAs and SDAs in the young mother centriole must wait for the next cell cycle.^28^ Thus, centrosome asymmetry occurs during mitotic exit (Figure 1).

In the context of ACD, centrosome asymmetry and inheritance patterns have been suggested to affect the distribution of cellular components that dictate the fate of daughter cells.^14^ Notably, recycling endosomes, which play a role in membrane trafficking and signaling, have emerged as key players in fate determination.^16^ Among these, CD133-positive recycling endosomes have garnered attention for their involvement in stemness and cellular differentiation pathways.^31^^,^^32^^,^^33^ CD133, also called prominin 1, was originally identified as a cell surface marker of human hematopoietic stem cells^34^^,^^35^ and mouse neuroepithelial cells.^36^ It was subsequently reported to function as a marker of cancer stem cells in many solid tumors, including NB.^37^ The CD133-positive cell population has a greater self-renewal ability and a stronger chemoresistance phenotype than the CD133-negative cell population. The expression of CD133 correlates with malignant characteristics and a poor prognosis in many tumors.^38^^,^^39^ While centrosome asymmetry and CD133-positive recycling endosomes have each been studied independently, their direct functional relationship during ACD remains unclear.

The present study aimed to elucidate the mechanisms underlying centrosome inheritance and its impact on the asymmetric distribution of CD133-positive recycling endosomes during cytokinesis in SK-N-DZ human NB cells. The results obtained showed that the inheritance of centrosomes adhered to specific rules in ACD: daughter cells inheriting centrosomes containing younger mother centrioles acquired a larger number of CD133-positive recycling endosomes, whereas those inheriting centrosomes with older mother centrioles acquired fewer. We also demonstrated that outer dense fiber protein 2 (Odf2)/cenexin,^25^ which localized to the DAs and SDAs of the centrosome,^40^^,^^41^ was essential for maintaining this asymmetric inheritance of CD133-positive recycling endosomes. The depletion of Odf2, which is necessary for the correct establishment of both appendages,^40^^,^^42^^,^^43^ led to the abnormally large distribution of recycling endosomes at both the centrosomes, effectively abolishing ACD.

The present results reveal a link between centrosome inheritance and the asymmetric allocation of recycling endosomes, providing fundamental insights into the molecular mechanisms that regulate ACD.

## Results

### Asymmetric distribution of centrosomal appendage proteins during cytokinesis in SK-N-DZ cells

Odf2 was originally identified as a molecular marker for centriole maturation^25^ and a scaffold protein that specifically localized to the DAs and SDAs of the mother centriole.^40^ A subsequent study demonstrated that Odf2 mainly localized to the SDAs as a scaffold protein for SDA formation^44^ and also localized to the base of DAs to support their 9-fold symmetry.^42^^,^^43^ Importantly, Odf2 remained localized to centrioles during the cell cycle.^20^^,^^21^ In addition, when Odf2 was used as a marker for the maturation of mother centrioles, a marked difference was detected in immunostaining signal intensities between mother and daughter centrosomes.^9^^,^^28^ Therefore, the distinction between mother and daughter centrosomes may be defined by the strength of the Odf2 signal during the final step of the cell cycle, cytokinesis.

**Figure 2:**
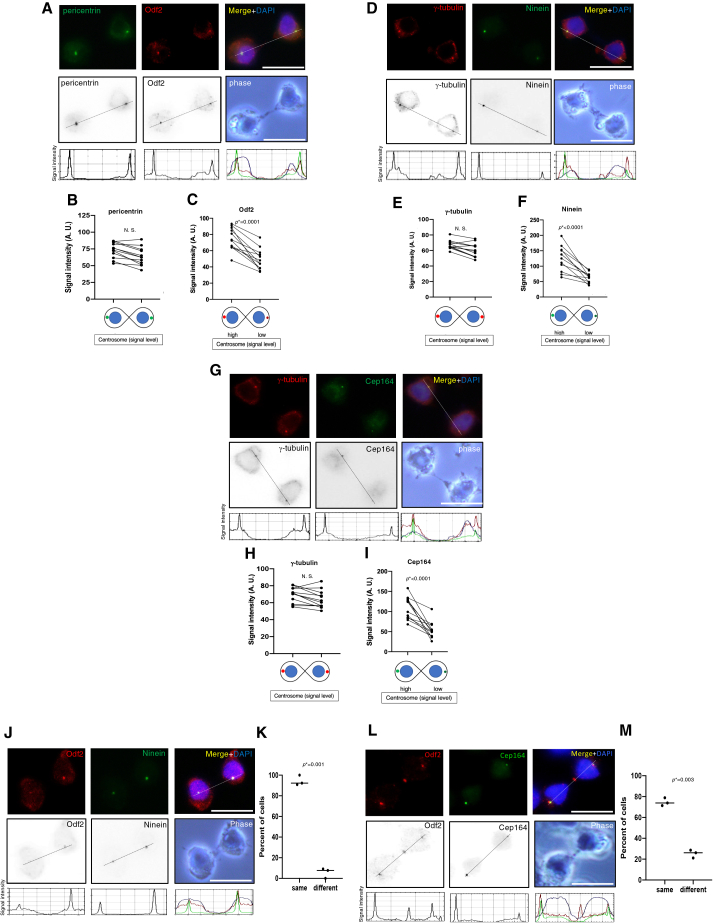
Centrosomal appendages show asymmetric (mature and immature) features during cytokinesis in SK-N-DZ cells (A) Representative immunostaining images of the subcellular localization of pericentrin and Odf2 during cytokinesis in SK-N-DZ cells. Pericentrin is green, Odf2 is red, and DAPI (DNA) is blue. Phase contrast images are also shown. Scale bars, 10 μm. Representative black and white images at the top are shown in the center. Plots show the signal intensities of pericentrin and Odf2 along the dotted lines. RGB profile plots also show the signal intensities of pericentrin, Odf2, and DAPI along the dotted lines of merged images. (B) The signal intensities of the cytokinesis pair of two pericentrin signals are shown as symbols and line graphs. Data are presented as mean ± SEM from *n* = 3 independent experiments; 12 cells were analyzed. Statistical significance was determined using a Student’s *t* test with equal variance (NS, no significance). (C) The signal intensities of the cytokinesis pair of two Odf2 signals are shown as symbols and line graphs. Data are presented as mean ± SEM from *n* = 3 independent experiments; 12 cells were analyzed. Statistical significance was determined using a Student’s *t* test with equal variance (*p* = 0.0001). (D) Representative immunostaining images of the subcellular localization of γ-tubulin and Ninein during cytokinesis in SK-N-DZ cells. γ-Tubulin is red, Ninein is green, and DAPI (DNA) is blue. Phase contrast images are also shown. Scale bars, 10 μm. Representative black and white images at the top are shown in the center. Plots show the signal intensities of γ-tubulin and Ninein along the dotted lines. RGB profile plots also show the signal intensities of γ-tubulin, Ninein, and DAPI along the dotted lines in the merged image. (E) The signal intensities of the cytokinesis pair of two γ-tubulin signals are shown as symbols and line graphs. Data are presented as mean ± SEM from *n* = 3 independent experiments; 12 cells were analyzed. Statistical significance was determined using a Student’s *t* test with equal variance (NS, no significance). (F) The signal intensities of the cytokinesis pair of two Ninein signals are shown as symbol and line graphs. Data are presented as mean ± SEM from *n* = 3 independent experiments; 12 cells were analyzed. Statistical significance was determined using a Student’s *t* test with equal variance (*p* < 0.0001). (G) Representative immunostaining images of the subcellular localization of γ-tubulin and Cep164 during cytokinesis in SK-N-DZ cells. γ-Tubulin is red, Cep164 is green, and DAPI (DNA) is blue. Phase contrast images are also shown. Scale bars, 10 μm. Representative black and white images at the top are shown in the center. Plots show the signal intensities of γ-tubulin and Cep164 along the dotted lines. RGB profile plots show the signal intensities of γ-tubulin, Cep164, and DAPI along the dotted line in the merged image. (H) The signal intensities of the cytokinesis pair of two γ-tubulin signals are shown as symbols and line graphs. Data are presented as mean ± SEM from *n* = 3 independent experiments; 12 cells were analyzed. Statistical significance was determined using a Student’s *t* test with equal variance (NS, no significance). (I) The signal intensities of the cytokinesis pair of two Cep164 signals are shown as symbol and line graphs. Data are presented as mean ± SEM from *n* = 3 independent experiments; 12 cells were analyzed. Statistical significance was determined using a Student’s *t* test with equal variance (*p* < 0.0001). (J) Representative immunostaining images of the subcellular localization of Odf2 and Ninein during cytokinesis in SK-N-DZ cells. Odf2 is red, Ninein is green, and DAPI (DNA) is blue. Phase contrast images are also shown. Scale bars, 10 μm. Representative black and white images are shown in the center. Plots show the signal intensities of Odf2 and Ninein along the dotted lines. RGB profile plots show the signal intensities of Odf2, Ninein, and DAPI along the dotted line in the merged image. (K) The signal intensities of the cytokinesis pair of Odf2 and Ninein signals are shown as dot graphs: “same” shows the high signal intensities of both Odf2 and Ninein; “different” shows the different signal intensities of Odf2 and Ninein. Data are presented as mean ± SEM from *n* = 3 independent experiments; 34 cells were analyzed. Statistical significance was determined using a Student’s *t* test with equal variance (*p* = 0.001). (L) Representative immunostaining images of the subcellular localization of Odf2 and Cep164 during cytokinesis in SK-N-DZ cells. Odf2 is red, Cep164 is green, and DAPI (DNA) is blue. Phase contrast images are also shown. Scale bars, 10 μm. Representative black and white images are shown in the center. Plots show the signal intensities of Odf2 and Cep164 along the dotted lines. RGB profile plots also show the signal intensities of Odf2, Cep164, and DAPI along the dotted line in the merged image. (M) The signal intensities of the cytokinesis pair of Odf2 and Cep164 signals are shown as dot graphs: “same” shows the high signal intensities of both Odf2 and Cep164; “different” shows the different signal intensities of Odf2 and Cep164. Data are presented as mean ± SEM from *n* = 3 independent experiments; 63 cells were analyzed. Statistical significance was determined using a Student’s *t* test with equal variance (*p* = 0.003).

We also examined the distribution of centrosomal appendage proteins, such as Odf2 as a marker of both DAs and SDAs,^40^^,^^41^^,^^45^ Ninein as a marker of SDAs,^46^^,^^47^ and Cep164 as a marker of DAs^20^^,^^48^ during cytokinesis in SK-N-DZ NB cells. Although Ninein and Cep164 are used as markers of the mother centriole during ACD in developing mouse brains,^49^^,^^50^ it is still unknown whether these SDAs and DAs show centrosome asymmetry in human NB cells. An immunofluorescence analysis revealed that while pericentrin and γ-tubulin staining showed similar signal intensities between daughter cells (Figures 2A, 2B, 2D, 2E, 2G, and 2H), Odf2, Ninein, and Cep164 showed asymmetric signal intensities characterized by mature and immature features (Figures 2A, 2C, 2D, 2F, 2G, and 2I). Specifically, one centrosome exhibited a mature state, while the other displayed an immature state. We also investigated whether the asymmetric signal intensity of Odf2 was consistent with that of Ninein and Cep164. We performed dual immunofluorescence staining experiments on Odf2 and Ninein or Cep164 during cytokinesis and compared signal intensities between mother and daughter centrosomes. When the centrosome with a higher signal intensity was the same for both Odf2 and Ninein or Cep164, it was categorized as “same.” Conversely, when the centrosome with a higher signal intensity differed between Odf2 and Ninein or Cep164, the classification was “different.” As shown in Figures 2J–2M, the signal intensities of Ninein and Cep164 were similar to that of Odf2. This asymmetry suggests a role for asymmetric centrosome inheritance during cell division in SK-N-DZ cells.

### Preferential localization of CD133-positive recycling endosomes to centrosomes with immature appendages

**Figure 3:**
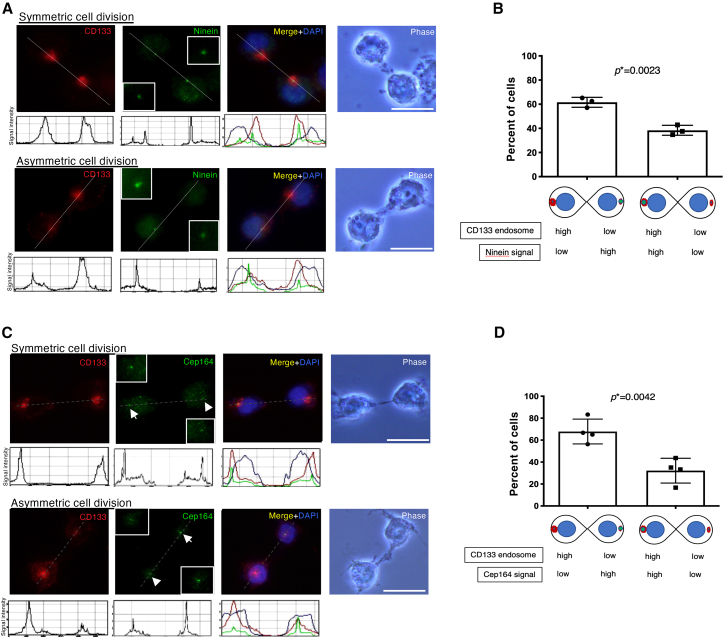
CD133-recycling endosomes preferentially localize to centrosomes with immature centrosome appendages (A) Representative immunostaining images of the cytokinesis of symmetric cell division or asymmetric cell division in SK-N-DZ cells. CD133 is red, Ninein is green, and DAPI (DNA) is blue. Insets show high magnification images of Ninein signals. Phase contrast images are also shown. Scale bars, 10 μm. Plots show the signal intensities of CD133 and Ninein along the dotted line. RGB profile plots also show the signal intensities of CD133, Ninein, and DAPI along the dotted line of the merged image. (B) Quantification of cells with the high or low signals of Ninein and the high or low signals of CD133 in the cytokinesis pair. Data are presented as mean ± SEM from *n* = 3 independent experiments; 45 cells were analyzed. Statistical significance was determined using a Student’s *t* test with equal variance (*p* = 0.0023). (C) Representative immunostaining images of the cytokinesis of symmetric cell division or asymmetric cell division in SK-N-DZ cells. CD133 is red, Cep164 is green, and DAPI (DNA) is blue. Insets show high magnification images of Cep164 signals. Phase contrast images are also shown. Scale bars, 10 μm. Plots show the signal intensities of CD133 and Cep164 along the dotted line. RGB profile plots also show the signal intensities of CD133, Cep164, and DAPI along the dotted line of the merged image. (D) Quantification of cells with the high or low signals of Cep164 and the high or low signals of CD133 in the cytokinesis pair. Data are presented as mean ± SEM from *n* = 3 independent experiments; 38 cells were analyzed. Statistical significance was determined using a Student’s *t* test with equal variance (*p* = 0.0042).

We examined the relationship between centrosomal maturity and the localization of CD133-positive recycling endosomes. Consistent with our recent findings, approximately 20% of the cell population showed an asymmetric CD133-recycling endosome distribution in SK-N-DZ cells (Figures S1A and S1B).^32^ The definition of ACD is described in the experimental model and study participant details section and in a recent study.^32^ Since another recycling endosome protein, Rab11, shows an asymmetric distribution during the final step of cell division in *Drosophila* sensory organ precursor cells,^51^ we investigated whether CD133-recycling endosomes showed a similar distribution of Rab11 during cytokinesis in SK-N-DZ cells. As expected, Rab11 also localized to centrosomes during cytokinesis in SK-N-DZ cells and showed a similar manner of localization to CD133-recycling endosomes in ACD (Figure S1C). Using fluorescent markers for CD133 and the centrosomal SDA protein, Ninein, we found that CD133-positive recycling endosomes preferentially localized to centrosomes with immature SDAs (Figures 3A and 3B). Similar results were obtained when Cep164, a DA protein, was used (Figures 3C and 3D). This selective association indicates a potential mechanism through which centrosome maturity affects the inheritance of endosomal components that are critical for cell properties. While we previously reported the asymmetric distribution of CD133-positive recycling endosomes,^32^ the present study demonstrates that this asymmetry correlates with centrosome maturity.

### Accumulation of CD133-and Rab11-positive recycling endosomes upon Odf2

#### Depletion

**Figure 4:**
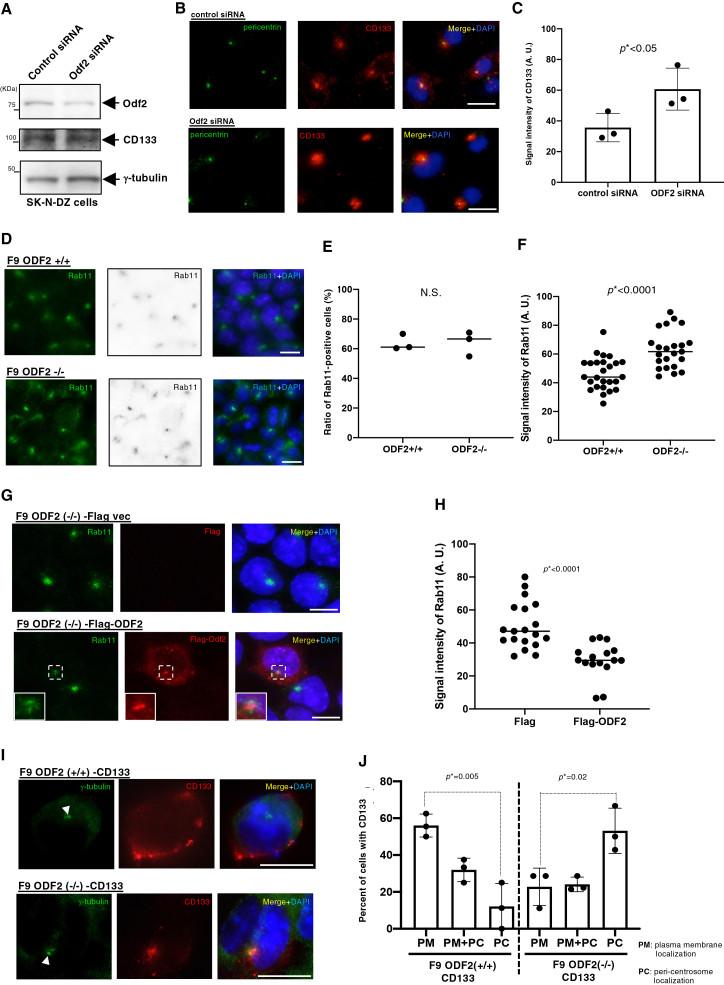
Depletion of Odf2 causes the accumulation of CD133-and Rab11-recycling endosomes around centrosomes (A) Immunoblot of Odf2 and CD133 in SK-N-DZ cells transfected with control or Odf2 siRNA. An immunoblot of γ-tubulin served as a loading control. (B) Representative images of pericentrin and CD133 in SK-N-DZ cells transfected with control or Odf2 siRNA. Pericentrin is green, CD133 is red, and DAPI (DNA) is blue. Scale bars, 10 μm. (C) Quantification of signal intensities of cells transfected with control or ODF2 siRNAs. Data are presented as mean ± SEM from *n* = 3 independent experiments; 124 cells (control siRNA) and 132 cells (Odf2 siRNA) were analyzed. Statistical significance was determined using a Student’s *t* test with equal variance (*p* < 0.05). (D) Representative images of Rab11 in F9 ODF2 (+/+) and F9 ODF2 (−/−) cells. Rab11 is green and DAPI (DNA) is blue. Scale bars, 10 μm. Black and white images show the signal intensities of Rab11. (E) Quantification of the distribution of Rab11 in F9 ODF2 (+/+) and F9 ODF2 (−/−) cells. Data are presented as mean ± SEM from *n* = 3 independent experiments; 122 F9 ODF2 (+/+) cells and 129 F9 ODF2 (−/−) cells were analyzed. Statistical significance was determined using a Student’s *t* test with equal variance (NS). (F) Quantification of the signal intensities of Rab11 in F9 ODF2 (+/+) and F9 ODF2 (−/−) cells. Data are presented as mean ± SEM from *n* = 3 independent experiments; 26 F9 ODF2 (+/+) cells and 23 F9 ODF2 (−/−) cells were analyzed. Statistical significance was determined using a Student’s *t* test with equal variance (*p* < 0.0001). (G) Representative images of Rab11 and Flag in F9 ODF2 (−/−) cells transfected with the Flag vector or Flag-Odf2 expression vector. Rab11 is green, Flag is red, and DAPI (DNA) is blue. Scale bars, 10 μm. (H) Quantification of the signal intensities of Rab11 in F9 ODF2 (−/−) cells transfected with the Flag vector or Flag-Odf2 expression vector. Data are presented as mean ± SEM from *n* = 3 independent experiments; 19 cells (Flag vector) and 16 cells (Flag-Odf2 vector) were analyzed. Statistical significance was determined using a Student’s *t* test with equal variance (*p* < 0.0001). (I) Representative images of γ-tubulin and CD133 in F9 ODF2 (+/+) cells transfected with the CD133 expression vector and F9 ODF2 (−/−) cells transfected with the CD133 expression vector. γ-Tubulin is green, CD133 is red, and DAPI (DNA) is blue. Scale bars, 10 μm. (J) Quantification of the subcellular distribution of CD133 in F9 ODF2 (+/+) cells transfected with the CD133 expression vector or F9 ODF2 (−/−) cells transfected with the CD133 expression vector. Data are presented as mean ± SEM from *n* = 3 independent experiments; 25 cells (F9 ODF2 (+/+)-CD133) and 37 cells (F9 ODF2 (−/−)-CD133) were analyzed. Statistical significance was determined using a Student’s *t* test with equal variance (*p* = 0.005 and *p* = 0.02, respectively).

Of the many appendage proteins of the centrosome, Odf2, which localizes to DAs and SDAs, plays essential roles both in the construction of SDAs and in ciliogenesis.^40^ Therefore, to examine the functional role of Odf2 in regulating the distribution of recycling endosomes, we used siRNA-mediated knockdown to deplete Odf2 in SK-N-DZ cells (Figures 4A and S2). While the protein expression level of CD133 did not change in Odf2 knockdown cells when compared with control siRNA cells (Figure 4A), immunofluorescence experiments revealed significant accumulation of CD133-positive recycling endosomes around centrosomes in Odf2-depleted cells (Figures 4B and 4C). Therefore, Odf2 appears to be crucial for the proper trafficking and spatial organization of recycling endosomes as previously described.^52^

We then used mouse teratocarcinoma F9 cells with the knockout of Odf2 (F9 ODF2(−/−) cells)^40^ to examine the behavior of recycling endosomes. Since endogenous CD133 expression was absent in F9 cells, we compared the distribution of Rab11-positive recycling endosomes in F9 cells with or without Odf2 expression. The results obtained showed that although Rab11-positive recycling endosomes localized to the pericentrosomal region in F9 ODF2(+/+) and F9 ODF2(−/−) cells with the same frequency, the signal intensity of Rab11 was slightly higher in F9 ODF2(−/−) cells than in F9 wild-type cells (Figures 4D–4F and S3). Therefore, we transfected a Flag-tagged ODF2 cDNA expression vector into F9 ODF2(−/−) cells and analyzed the signal intensity of Rab11. As expected, the transfection of the ODF2 expression vector reduced the signal intensity of Rab11-positive recycling endosomes (Figures 4G and 4H). These results suggest that Odf2 promptly controlled the turnover of recycling endosomes between the plasma membrane and centrosomes.^52^ We then transfected a CD133 cDNA expression vector into F9 ODF2 (+/+) and ODF2(−/−) cells and analyzed the localization pattern of CD133 in these cells using immunofluorescent imaging. While CD133 endosomes mainly localized to the plasma membrane in F9 ODF2 (+/+) cells, they mainly localized to the pericentrosomal region in F9 ODF2(−/−) cells (Figures 4I and 4J). These results suggest that Odf2 controls the localization pattern of CD133-positive recycling endosomes and the loss of mature Odf2 caused the pericentrosomal localization of CD133.

### Odf2 knockdown suppresses asymmetric inheritance and promotes SCD

**Figure 5:**
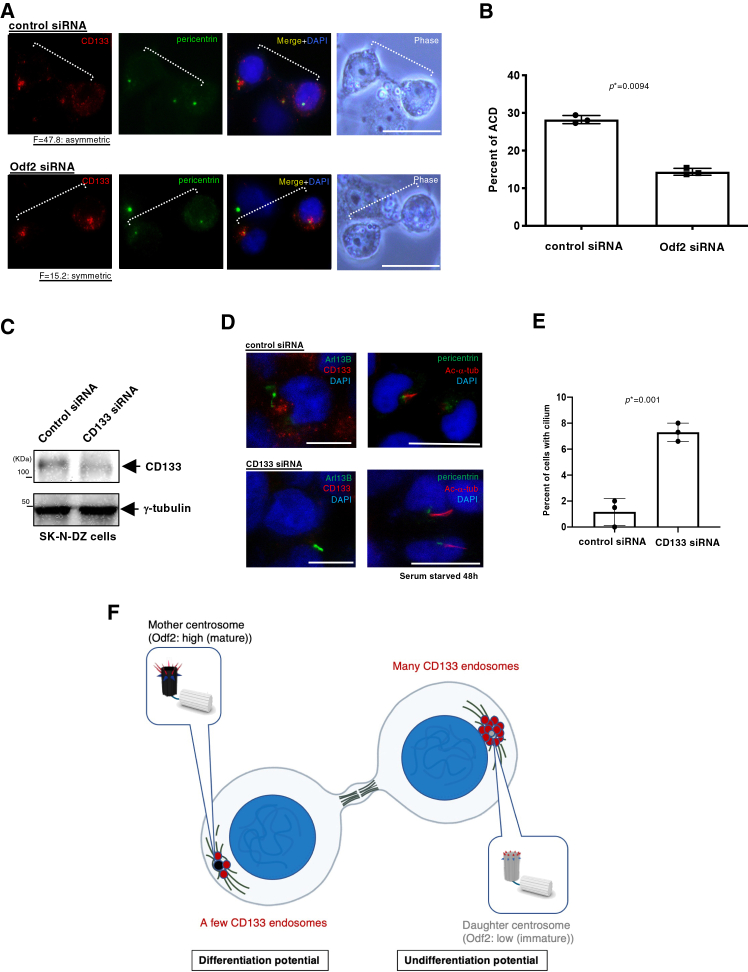
Knockdown of Odf2 suppresses the asymmetric inheritance of CD133-recycling endosomes, resulting in symmetric cell division in SK-N-DZ cells (A) Representative images of CD133 and pericentrin in SK-N-DZ cells transfected with control or Odf2 siRNA. CD133 is red, Pericentrin is green, and DAPI (DNA) is blue. Phase contrast images are also shown. Scale bars, 10 μm. (B) Quantification of the ratio of asymmetric cell division in SK-N-DZ cells transfected with control or ODF2 siRNAs. Data are presented as mean ± SEM from *n* = 3 independent experiments; 39 cells (control siRNA) and 35 cells (Odf2 siRNA) were analyzed. Statistical significance was determined using a Student’s *t* test with equal variance (*p* = 0.0094). (C) Immunoblot of CD133 in SK-N-DZ cells transfected with control or CD133 siRNA. An immunoblot of γ-tubulin served as a loading control. (D) Representative images of CD133 and Arl13B or pericentrin and acetylated-α-tubulin (AC-α-tub) in SK-N-DZ cells transfected with control or CD133 siRNA. Transfected cells were subjected to serum starvation for 48 h Arl13B is green, CD133 is red, and DAPI (DNA) is blue (left). Pericentrin is green, AC-α-tub is red, and DAPI (DNA) is blue (right). Scale bars, 10 μm. (E) Quantification of the ratio of primary cilia in SK-N-DZ cells transfected with control or CD133 siRNAs. Data are presented as mean ± SEM from *n* = 3 independent experiments; 173 cells (control siRNA) and 179 cells (CD133 siRNA) were analyzed. Statistical significance was determined using a Student’s *t* test with equal variance (*p* = 0.0048). (F) Schematic image of asymmetric cell division in SK-N-DZ cells in the present study.

Although Odf2 is known to regulate centrosomal appendage formation, its role in asymmetric organelle inheritance during ACD has not been established. We, therefore, examined the impact of the knockdown of Odf2 on the inheritance of CD133-positive recycling endosomes and cell division outcomes. In control cells, the asymmetric inheritance of CD133-positive recycling endosomes was observed, which was consistent with the frequency of ACD (approximately 20%) of parental SK-N-DZ cells (Figure 5A). However, in Odf2-depleted cells, the inheritance pattern became symmetric, with an almost equal distribution of CD133-positive recycling endosomes to both daughter cells (Figure 5A). The quantitative analysis demonstrated that the knockdown of Odf2 significantly reduced the percentage of ACD, resulting in a higher frequency of symmetric divisions (Figure 5B).

The physiological meaning of the ACD of CD133-positive recycling endosomes in SK-N-DZ cells remains unclear; therefore, we examined this issue. We used siRNA-mediated knockdown to deplete CD133 and analyzed the formation of primary cilia as a marker of cell differentiation in SK-N-DZ cells (Figures 5C and S4). Cancer cell lines are generally resistant to serum starvation; however, in SK-N-DZ cells, a small percentage of cells appeared to undergo G1/G0 arrest (Figure S4). Immunofluorescence experiments subsequently revealed that a significant siRNA-mediated reduction in CD133-positive recycling endosomes caused primary ciliogenesis (Figures 5D and 5E). Therefore, CD133-positive recycling endosomes at the centrosome induced an undifferentiated cell fate. One of the physiological functions of ACD in the present study was the regulation of cell fate; ACD consistently maintained two cell populations among cells with a differentiation potential and undifferentiation potential.

Collectively, the present results demonstrated that centrosomal appendages exhibited asymmetry during cytokinesis, which affected the localization of CD133-positive recycling endosomes. The centrosomal protein, Odf2, is essential for maintaining this asymmetry because its depletion disrupts endosome localization and leads to SCD. These results suggest a critical role for centrosome-associated proteins in the regulation of cell fate decisions in NB cells (Figure 5F).

## Discussion

The present study provides insights into the critical role of centrosome inheritance and CD133-positive recycling endosomes in the context of ACD in SK-N-DZ human NB cells. First, we demonstrate a direct correlation between centrosome age and the asymmetric distribution of CD133-positive recycling endosomes. Second, we show that Odf2 is required for maintaining this asymmetry during cytokinesis. Third, we propose a functional link between centrosome inheritance and endosome-mediated cell fate regulation. These results obtained suggest that the proper segregation of these organelles is fundamental to maintaining the balance between self-renewal and differentiation. A compelling link was revealed between the structural components of the centrosome and the fate-determining mechanisms mediated by recycling endosomes. One of the key observations is that daughter cells inheriting centrosomes containing younger mother centrioles consistently receive a larger number of CD133-positive recycling endosomes (Figure 5F). This asymmetry is important because CD133, a well-established marker of cancer stem cells,^33^^,^^38^^,^^39^ is intimately involved in signaling pathways that promote stem-like properties and tumor-initiating capacity. In addition, CD133-positive recycling endosomes localized to centrosomes and suppressed primary ciliogenesis.^31^ This result highlights the control of cell division fates by CD133-positive recycling endosomes around centrosomes. Therefore, the preferential distribution of CD133-positive recycling endosomes to one daughter cell implies a mechanism by which ACD not only creates cellular diversity but also preserves a population of cells with stem-like traits.

Centrosome inheritance in ACD was originally discovered in *Drosophila* male germ stem cells by Yamashita et al.^53^ Their findings showed that while the mother centrosome was inherited by cells with stem cell properties, the daughter centrosome was inherited by cells with differentiation potential. Wang et al. reported similar findings by using mouse neuronal progenitor cells.^50^ Habib et al. also obtained similar findings by using mouse embryonic stem cells.^54^ On the other hand, Yamashita’s group demonstrated that while the daughter centrosome was inherited by cells with stem cell properties, the mother centrosome was inherited by cells with differentiation potential in *Drosophila* female germ stem cells.^55^ Januschke et al. obtained similar findings by using *Drosophila* neuroblast systems.^56^ Our previous findings^9^ and the present results collectively indicate that the human NB cell system corresponds to *Drosophila* female germ stem cells and *Drosophila* neuroblast systems. However, further studies are needed to resolve this issue.

The mechanisms by which centrosome inheritance affects the distribution of recycling endosomes remain an area of great interest. The present results indicate that Odf2 plays a pivotal role in regulating this process. Odf2, which localized to centrosomal DAs and SDAs, appeared to be necessary for maintaining the asymmetric partitioning of recycling endosomes. The depletion of Odf2 resulted in the marked loss of ACD, as evidenced by the distribution of recycling endosomes at both centrosomes. This aberrant distribution disrupts the normal asymmetry required for cell fate decisions and may emphasize the importance of centrosome-associated proteins in orchestrating organelle inheritance. The loss of ACD upon the depletion of Odf2 raises questions about the underlying molecular mechanisms. Odf2 may play a role in signaling pathways that regulate endosomal trafficking. A previous study demonstrated that Odf2 interacted with Rab11-GTP, but not Rab11-GDP at centrosomes^52^ and also that the depletion of Odf2 or Evi5, a Rab11 GTPase-activating protein, increased centrosomal Rab11-positive endosomes.^52^ Moreover, Rab11 was recently shown to maintain an undifferentiated state in *Drosophila* larva and midgut precursors.^57^ Therefore, future research will be necessary to elucidate these mechanisms and clarify how Odf2 integrates with known regulators of ACD.

The present results provide insights into the broader implications of centrosome-mediated organelle inheritance in cancer. Tumor heterogeneity by ACD is considered to be a hallmark of many cancers,^6^ contributing to tumor progression and resistance to conventional therapies.^6^ By elucidating the mechanisms by which centrosome dynamics affect the distribution of stem cell markers, such as CD133, we may obtain a more detailed understanding of the cellular and molecular mechanisms underlying tumorigenesis. Our results indicate the potential of targeting centrosome-associated proteins, such as Odf2, as a viable strategy to change cell fate outcomes and potentially hinder the self-renewal capacity of cancer stem cells.

### Limitations of the study

There are some limitations that need to be addressed. The use of a single NB cell line, SK-N-DZ, necessitates further investigations across different models to confirm the universality of these mechanisms. Additionally, while our results implicate Odf2 as a regulator of ACD, the precise pathways through which it operates remain to be elucidated. Future studies will be invaluable for delineating the temporal and spatial dynamics of centrosome and endosome inheritance in ACD.

## Resource availability

### Lead contact

Further information and requests for resources and reagents should be directed to and will be fulfilled by the lead contact, Hideki Izumi (izumi-hideki@koseikan.jp).

### Materials availability

This study did not generate new unique reagents. Materials described in this study are available from the lead contact upon reasonable request.

### Data and code availability

• Data reported in this paper will be shared by the lead contact upon request.
• This paper does not report original code.
• Any additional information required to reanalyze the data reported in this paper are available from the lead contact upon request.
• No custom code was generated in this study.

## Acknowledgments

We are grateful to Dr. E. Sadashima for the advice on statistical analyses, S. Tsuchida for technical assistance, and I. Tanaka, M. Shimajiri, and M. Ureshino for secretarial assistance. We also thank our laboratory members for their continuous encouragement.

## Author contributions

H.I. conceived and designed the experiments; H.I. performed the majority of the experiments; T.M. performed some experiments; H.I., T.M., S.T., and A.N. analyzed the data; H.I. wrote the manuscript; H.I. and A.N. contributed to the research design and edited the manuscript. All authors read and approved the final manuscript.

## Declaration of interests

The authors declare no competing interests.

## Declaration of generative AI and AI-assisted technologies in the writing process

The authors did not use generative AI or AI-assisted technologies in the preparation of this manuscript.

## STAR★Methods

### Key resources table

**Table:** 

REAGENT or RESOURCE	SOURCE	IDENTIFIER
**Antibodies**		

CD133 (AC133)	Miltenyi Biotec	Cat# 130-090-422; RRID: AB_244339
CD133	Cell Signaling Technology	Cat# 64326; RRID: AB_2721172
Acetylated-α-Tubulin	Cell Signaling Technology	Cat# 5335; RRID: AB_10544694
Acetylated-α-Tubulin	Sigma-Aldrich	Cat# T7451; RRID: AB_609894
γ-Tubulin (clone GTU88)	Sigma-Aldrich	Cat# T6557; RRID: AB_477584
γ-Tubulin	Sigma-Aldrich	Cat# T5192; RRID: AB_261690
Rab11	Cell Signaling Technology	Cat#5589; RRID: AB_10693925
Pericentrin	Abcam	Cat# Ab4448; RRID: AB_304461
Flag	Sigma-Aldrich	Cat# F1804; RRID: AB_262044
Flag	Cell Signaling Technology	Cat# 2368; RRID: AB_2217020
Odf2	Abnova	Cat# H00004957-M01; RRID: AB_1137338
Odf2	Sigma-Aldrich	Cat# HPA001874; RRID: AB_1079522
Ninein	Sigma-Aldrich	Cat# HPA005939
Cep164	Sigma-Aldrich	Cat# HPA037605; RRID: AB_10672908
Arl13B	ProteinTech	Cat# 17711-1-AP; RRID: AB_2060867

**Chemicals, peptides, and recombinant proteins**		

Triton X-100	WAKO	Cat# 807423
NP-40	WAKO	Cat# 144-08311
DMSO	WAKO	Cat# 047-29353
DAPI	Sigma-Aldrich	Cat# D9542
DMEM	Gibco	Cat# 11995-073
Lipofectamine 3000	ThermoFisher Scientific	Cat# L3000008
Lipofectamine RNAiMAX	ThermoFisher Scientific	Cat# 13778075
Anti-anti	Gibco	Cat# 15240-096
FBS	Gibco	Cat# 10437-028
ECL prime	Cytiva	Cat# RPN2232

**Experimental models: Cell lines**		

SK-N-DZ	ATCC	Cat# CRL-2149
F9	RIKEN	Cat# RCB1555
F9 ODF2 KO	RIKEN	Cat# RCB2808

**Experimental models: Organisms/strains**		

DH5α	TOYOBO	Cat# DNA-913

**Oligonucleotides**		

Control siRNA	ThermoFisher Scientific	Cat# AM4611
CD133 siRNA	ThermoFisher Scientific	Cat# S16880
ODF2 siRNA	ThermoFisher Scientific	Cat# S9827

**Recombinant DNA**		

pCMV-Flag	Origene	Cat# PS100001
pcDNA	ThermoFisher Scientific	Cat# V79020
pcDNA-CD133 (WT)	Genscript	N/A
pcDNA-Flag-ODF2	Genscript	N/A

**Others**		

Muse Cell Analyzer	Merck	0500-3115B
Muse Cell cycle kit	Merck	MCH100106

**Software and algorithms**		

Prism8	GraphPad	https://www.graphpad.com/scientific-software/prism/
Photoshop 2020	Adobe	https://www.adobe.com
ImageJ	NIH	https://imagej.nih.gov/ij/

### Experimental model and study participant details

#### Cell culture and transfection

SK-N-DZ cells were obtained from the American Type Culture Collection. F9 cells and Odf2-knockout F9 cells were obtained from RIKEN Cell Bank (BRC). All cell lines were validated by a short tandem repeat analysis. These cells were maintained in complete medium, consisting of Dulbecco’s modified Eagle’s medium, supplemented with 10% fetal bovine serum, antibiotic-antimycotic solution in an atmosphere containing 5% CO_2_ at 37°C. Lipofectamine 3000 was used for plasmid transient transfection according to the manufacturer’s instructions. Cells were analyzed 20 h after transfection. Regarding RNA interference, siRNA duplexes were transfected using Lipofectamine RNAiMAX as described by the manufacturer.

### Method details

#### Plasmids

The pCMV-Flag vector (PS100001) was obtained from OriGene Technologies. pcDNA 3.1 (V79020) was purchased from ThermoFisher Scientific. Full-length ODF2 and CD133 cDNAs were purchased from GenScript. cDNAs were confirmed by DNA sequencing.

#### Indirect immunofluorescence cell imaging

Cells grown on coverslips were briefly washed in phosphate-buffered saline (PBS) three times and were then fixed with 4% formaldehyde solution at room temperature (RT) or ice cold methanol for 20 min. Cells were treated with 1% NP-40 in PBS solution for 10 min and then incubated with blocking solution (15% bovine serum albumin in PBS) for 1 h. Cells were then probed with primary antibodies at 37°C for 1 h, and antibody-antigen complexes were detected using an Alexa Fluor 594- or Alexa Fluor 488-conjugated donkey secondary antibody after an incubation at RT for 1 h. Samples were washed three times with Tris-buffered saline (TBS) after each incubation and then counterstained with 4’, 6’-diamidino-2-phenylindole (DAPI). Immunostained cells were examined under a fluorescence microscope (Olympus IX73, Tokyo, Japan) using a 100× or 60× objective lens. Fluorescence images were captured with a CCD camera (Olympus, DP27), and processed with Adobe Photoshop 2020 and ImageJ.

#### Immunoblotting

Cells were lysed in SDS/Nonidet P-40 lysis buffer (1% SDS, 1% Nonidet P-40, 50 mM Tris [pH 8.0], 150 mM NaCl, 2 μg/ml of leupeptin, 2 μg/ml of aprotinin, 1 mM phenylmethylsulfonyl fluoride [PMSF], 5 mM NaF, and 100 μM Na_3_VO_4_). Lysates were boiled for 5 min and then cleared by centrifugation at 15,000 rpm at 4°C. The protein concentration of the supernatant was measured using the Bradford protein assay reagent (BioRad), as previously reported. Lysates were boiled for 5 min in sample buffer. Samples were then resolved by SDS-PAGE and transferred onto Immobilon-P (Millipore Corp.) membranes. Blots were initially incubated in blocking buffer (5% [w/v] non-fat dry milk in TBS plus 0.05% Tween 20) for 1 h. They were then incubated with a primary antibody at 4°C for 16 h, followed by an incubation with a horseradish peroxidase-conjugated secondary antibody at RT for 1 h. The antibody-antigen complex was visualized by ECL-prime chemiluminescence.

#### Flow cytometry

The percentage of SK-N-DZ cells transfected with control siRNA and CD133 siRNA with or without 10% serum for 48 h in G0/G1, G2/M, and S phases was estimated by triplicate measurements using a FACS analysis (Muse Cell Analyzer) and the Muse cell-cycle analysis program.

### Quantification and statistical analysis

All cell images were quantified using Image J and Adobe Photoshop 2020. All statistical analyses were performed using Graphpad Prism 8. Experimental groups were compared using an unpaired Student’s *t*-test due to the binary nature of the datasets. Probability values less than 0.05 were considered to be significant and data are presented as the mean ± standard error of the mean. Image J was used to measure the fluorescence intensity of staining for the two dividing daughter cells. To classify the cell division type as symmetrical or asymmetrical, the percent difference in fluorescence intensity between daughter cells (F) was calculated as follows: (F1-F2)/(F1+F2) × 100; where F1 and F2 represent the fluorescence values of staining by a given antibody for the two resulting daughter cells. After obtaining the percent (%) deviation for each dividing pair, each cell division was categorized as symmetrical or asymmetrical using the 25% difference cut-off value for CD133.

## References

[1] Gönczy P. (2008). Mechanisms of asymmetric cell division: flies and worms pave the way. Nat. Rev. Mol. Cell Biol..

[2] Knoblich J.A. (2010). Asymmetric cell division: recent developments and their implications for tumour biology. Nat. Rev. Mol. Cell Biol..

[3] Caussinus E., Gonzalez C. (2005). Induction of tumor growth by altered stem-cell asymmetric division in Drosophila melanogaster. Nat. Genet..

[4] Clevers H. (2005). Stem cells, asymmetric division and cancer. Nat. Genet..

[5] Basto R., Brunk K., Vinadogrova T., Peel N., Franz A., Khodjakov A., Raff J.W. (2008). Centrosome amplification can initiate tumorigenesis in flies. Cell.

[6] Li Z., Zhang Y.Y., Zhang H., Yang J., Chen Y., Lu H. (2022). Asymmetric Cell Division and Tumor Heterogeneity. Front. Cell Dev. Biol..

[7] Brodeur G.M. (2003). Neuroblastoma: biological insights into a clinical enigma. Nat. Rev. Cancer.

[8] Matthay K.K., Maris J.M., Schleiermacher G., Nakagawara A., Mackall C.L., Diller L., Weiss W.A. (2016). Neuroblastoma. Nat. Rev. Dis. Primers.

[9] Izumi H., Kaneko Y. (2012). Evidence of asymmetric cell division and centrosome inheritance in human neuroblastoma cells. Proc. Natl. Acad. Sci. USA.

[10] Izumi H., Kaneko Y. (2014). Trim32 facilitates degradation of MYCN on spindle poles and induces asymmetric cell division in human neuroblastoma cells. Cancer Res..

[11] Izumi H., Kaneko Y., Nakagawara A. (2021). Analysis of Asymmetric Cell Division Using Human Neuroblastoma Cell Lines as a Model System. Symmetry.

[12] Ross R.A., Spengler B.A. (2007). Human neuroblastoma stem cells. Semin. Cancer Biol..

[13] Izumi H., Kaneko Y., Nakagawara A. (2020). The Role of MYCN in Symmetric vs. Asymmetric Cell Division of Human Neuroblastoma Cells. Front. Oncol..

[14] Chen C., Yamashita Y.M. (2021). Centrosome-centric view of asymmetric stem cell division. Open Biol..

[15] Fürthauer M., González-Gaitán M. (2009). Endocytosis and mitosis: a two-way relationship. Cell Cycle.

[16] Daeden A., Gonzalez-Gaitan M. (2018). Endosomal Trafficking During Mitosis and Notch-Dependent Asymmetric Division. Prog. Mol. Subcell. Biol..

[17] Azimzadeh J., Bornens M. (2007). Structure and duplication of the centrosome. J. Cell Sci..

[18] Uzbekov R.E., Avidor-Reiss T. (2020). Principal Postulates of Centrosomal Biology. Version 2020. Cells.

[19] Ma D., Wang F., Teng J., Huang N., Chen J. (2023). Structure and function of distal and subdistal appendages of the mother centriole. J. Cell Sci..

[20] Viol L., Hata S., Pastor-Peidro A., Neuner A., Murke F., Wuchter P., Ho A.D., Giebel B., Pereira G. (2020). Nek2 kinase displaces distal appendages from the mother centriole prior to mitosis. J. Cell Biol..

[21] Bowler M., Kong D., Sun S., Nanjundappa R., Evans L., Farmer V., Holland A., Mahjoub M.R., Sui H., Loncarek J. (2019). High-resolution characterization of centriole distal appendage morphology and dynamics by correlative STORM and electron microscopy. Nat. Commun..

[22] Schmidt K.N., Kuhns S., Neuner A., Hub B., Zentgraf H., Pereira G. (2012). Cep164 mediates vesicular docking to the mother centriole during early steps of ciliogenesis. J. Cell Biol..

[23] Kong D., Farmer V., Shukla A., James J., Gruskin R., Kiriyama S., Loncarek J. (2014). Centriole maturation requires regulated Plk1 activity during two consecutive cell cycles. J. Cell Biol..

[24] Vorobjev I.A., Chentsov Yu S. (1982). Centrioles in the cell cycle. I. Epithelial cells. J. Cell Biol..

[25] Lange B.M., Gull K. (1995). A molecular marker for centriole maturation in the mammalian cell cycle. J. Cell Biol..

[26] Tanos B.E., Yang H.J., Soni R., Wang W.J., Macaluso F.P., Asara J.M., Tsou M.F.B. (2013). Centriole distal appendages promote membrane docking, leading to cilia initiation. Genes Dev..

[27] Uzbekov R., Alieva I. (2018). Who are you, subdistal appendages of centriole?. Open Biol..

[28] Anderson C.T., Stearns T. (2009). Centriole age underlies asynchronous primary cilium growth in mammalian cells. Curr. Biol..

[29] Thomas A., Meraldi P. (2024). Centrosome age breaks spindle size symmetry even in cells thought to divide symmetrically. J. Cell Biol..

[30] Nigg E.A., Holland A.J. (2018). Once and only once: mechanisms of centriole duplication and their deregulation in disease. Nat. Rev. Mol. Cell Biol..

[31] Izumi H., Li Y., Shibaki M., Mori D., Yasunami M., Sato S., Matsunaga H., Mae T., Kodama K., Kamijo T. (2019). Recycling endosomal CD133 functions as an inhibitor of autophagy at the pericentrosomal region. Sci. Rep..

[32] Izumi H., Li Y., Yasunami M., Sato S., Mae T., Kaneko Y., Nakagawara A. (2022). Asymmetric Pericentrosomal CD133 Endosomes Induce the Unequal Autophagic Activity During Cytokinesis in CD133-Positive Human Neuroblastoma Cells. Stem Cell..

[33] Pleskač P., Fargeas C.A., Veselska R., Corbeil D., Skoda J. (2024). Emerging roles of prominin-1 (CD133) in the dynamics of plasma membrane architecture and cell signaling pathways in health and disease. Cell. Mol. Biol. Lett..

[34] Miraglia S., Godfrey W., Yin A.H., Atkins K., Warnke R., Holden J.T., Bray R.A., Waller E.K., Buck D.W. (1997). A novel five-transmembrane hematopoietic stem cell antigen: isolation, characterization, and molecular cloning. Blood.

[35] Yin A.H., Miraglia S., Zanjani E.D., Almeida-Porada G., Ogawa M., Leary A.G., Olweus J., Kearney J., Buck D.W. (1997). AC133, a novel marker for human hematopoietic stem and progenitor cells. Blood.

[36] Weigmann A., Corbeil D., Hellwig A., Huttner W.B. (1997). Prominin, a novel microvilli-specific polytopic membrane protein of the apical surface of epithelial cells, is targeted to plasmalemmal protrusions of non-epithelial cells. Proc. Natl. Acad. Sci. USA.

[37] Takenobu H., Shimozato O., Nakamura T., Ochiai H., Yamaguchi Y., Ohira M., Nakagawara A., Kamijo T. (2011). CD133 suppresses neuroblastoma cell differentiation via signal pathway modification. Oncogene.

[38] Grosse-Gehling P., Fargeas C.A., Dittfeld C., Garbe Y., Alison M.R., Corbeil D., Kunz-Schughart L.A. (2013). CD133 as a biomarker for putative cancer stem cells in solid tumours: limitations, problems and challenges. J. Pathol..

[39] Li Z. (2013). CD133: a stem cell biomarker and beyond. Exp. Hematol. Oncol..

[40] Ishikawa H., Kubo A., Tsukita S., Tsukita S. (2005). Odf2-deficient mother centrioles lack distal/subdistal appendages and the ability to generate primary cilia. Nat. Cell Biol..

[41] Tateishi K., Yamazaki Y., Nishida T., Watanabe S., Kunimoto K., Ishikawa H., Tsukita S. (2013). Two appendages homologous between basal bodies and centrioles are formed using distinct Odf2 domains. J. Cell Biol..

[42] Chong W.M., Wang W.J., Lo C.H., Chiu T.Y., Chang T.J., Liu Y.P., Tanos B., Mazo G., Tsou M.F.B., Jane W.N. (2020). Super-resolution microscopy reveals coupling between mammalian centriole subdistal appendages and distal appendages. eLife.

[43] Chang T.J.B., Hsu J.C.C., Yang T.T. (2023). Single-molecule localization microscopy reveals the ultrastructural constitution of distal appendages in expanded mammalian centrioles. Nat. Commun..

[44] Huang N., Xia Y., Zhang D., Wang S., Bao Y., He R., Teng J., Chen J. (2017). Hierarchical assembly of centriole subdistal appendages via centrosome binding proteins CCDC120 and CCDC68. Nat. Commun..

[45] Kashihara H., Chiba S., Kanno S.I., Suzuki K., Yano T., Tsukita S. (2019). Cep128 associates with Odf2 to form the subdistal appendage of the centriole. Genes Cells.

[46] Mogensen M.M., Malik A., Piel M., Bouckson-Castaing V., Bornens M. (2000). Microtubule minus-end anchorage at centrosomal and non-centrosomal sites: the role of ninein. J. Cell Sci..

[47] Chen C.H., Howng S.L., Cheng T.S., Chou M.H., Huang C.Y., Hong Y.R. (2003). Molecular characterization of human ninein protein: two distinct subdomains required for centrosomal targeting and regulating signals in cell cycle. Biochem. Biophys. Res. Commun..

[48] Graser S., Stierhof Y.D., Lavoie S.B., Gassner O.S., Lamla S., Le Clech M., Nigg E.A. (2007). Cep164, a novel centriole appendage protein required for primary cilium formation. J. Cell Biol..

[49] Paridaen J.T.M.L., Wilsch-Bräuninger M., Huttner W.B. (2013). Asymmetric inheritance of centrosome-associated primary cilium membrane directs ciliogenesis after cell division. Cell.

[50] Wang X., Tsai J.W., Imai J.H., Lian W.N., Vallee R.B., Shi S.H. (2009). Asymmetric centrosome inheritance maintains neural progenitors in the neocortex. Nature.

[51] Emery G., Hutterer A., Berdnik D., Mayer B., Wirtz-Peitz F., Gaitan M.G., Knoblich J.A. (2005). Asymmetric Rab 11 endosomes regulate delta recycling and specify cell fate in the Drosophila nervous system. Cell.

[52] Hehnly H., Chen C.T., Powers C.M., Liu H.L., Doxsey S. (2012). The centrosome regulates the Rab11- dependent recycling endosome pathway at appendages of the mother centriole. Curr. Biol..

[53] Yamashita Y.M., Mahowald A.P., Perlin J.R., Fuller M.T. (2007). Asymmetric inheritance of mother versus daughter centrosome in stem cell division. Science.

[54] Habib S.J., Chen B.C., Tsai F.C., Anastassiadis K., Meyer T., Betzig E., Nusse R. (2013). A localized Wnt signal orients asymmetric stem cell division in vitro. Science.

[55] Salzmann V., Chen C., Chiang C.Y.A., Tiyaboonchai A., Mayer M., Yamashita Y.M. (2014). Centrosome-dependent asymmetric inheritance of the midbody ring in Drosophila germline stem cell division. Mol. Biol. Cell.

[56] Januschke J., Llamazares S., Reina J., Gonzalez C. (2011). Drosophila neuroblasts retain the daughter centrosome. Nat. Commun..

[57] Pandey A., Roy J.K. (2024). Rab11 maintains the undifferentiated state of adult midgut precursors via DPP pathway. Exp. Cell Res..

