# Supplementary material for: The Odf2 status contributes to asymmetric inheritance of CD133-positive recycling endosomes during cytokinesis in SK-N-DZ human neuroblastoma cells
Source: iScience. 2026 Aug 6;29(8):117062. doi: 10.1016/j.isci.2026.117062 (PMC13475681; doi:10.1016/j.isci.2026.117062)
Supplement: Supplementary file 1 [file mmc1.pdf]

iScience, Volume *29*

Supplemental information

The Odf2 status contributes to asymmetric inheritance of CD133-positive recycling endosomes during cytokinesis in SK-N-DZ human neuroblastoma cells

Hideki Izumi, Takao Mae, Sachiko Tsukita, and Akira Nakagawara

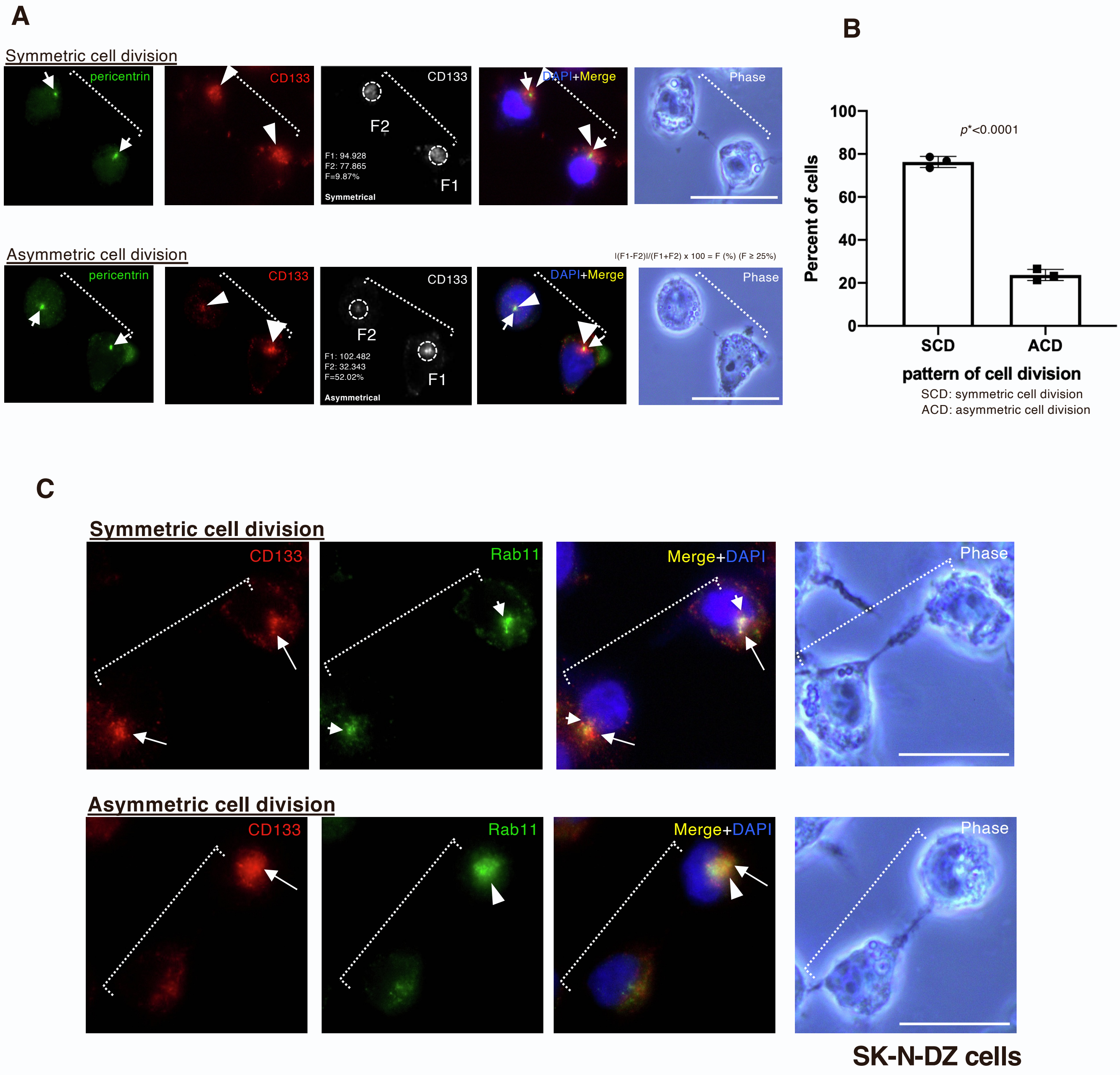

A
Symmetric cell division
pericentrin
CD133
CD133
F2
F1
F1: 94.928
F2: 77.865
F=9.87%
Symmetrical
DAPI+Merge
Phase
Asymmetric cell division
I(F1-F2)I/(F1+F2) x 100 = F (%) (F ≥ 25%)
pericentrin
CD133
CD133
F2
F1
F1: 102.482
F2: 32.343
F=52.02%
Asymmetrical
DAPI+Merge
Phase
B
p*<0.0001
Percent of cells
100
80
60
40
20
0
SCD
ACD
pattern of cell division
SCD: symmetric cell division
ACD: asymmetric cell division
C
Symmetric cell division
CD133
Rab11
Merge+DAPI
Phase
Asymmetric cell division
CD133
Rab11
Merge+DAPI
Phase
SK-N-DZ cells

Figure S1

A

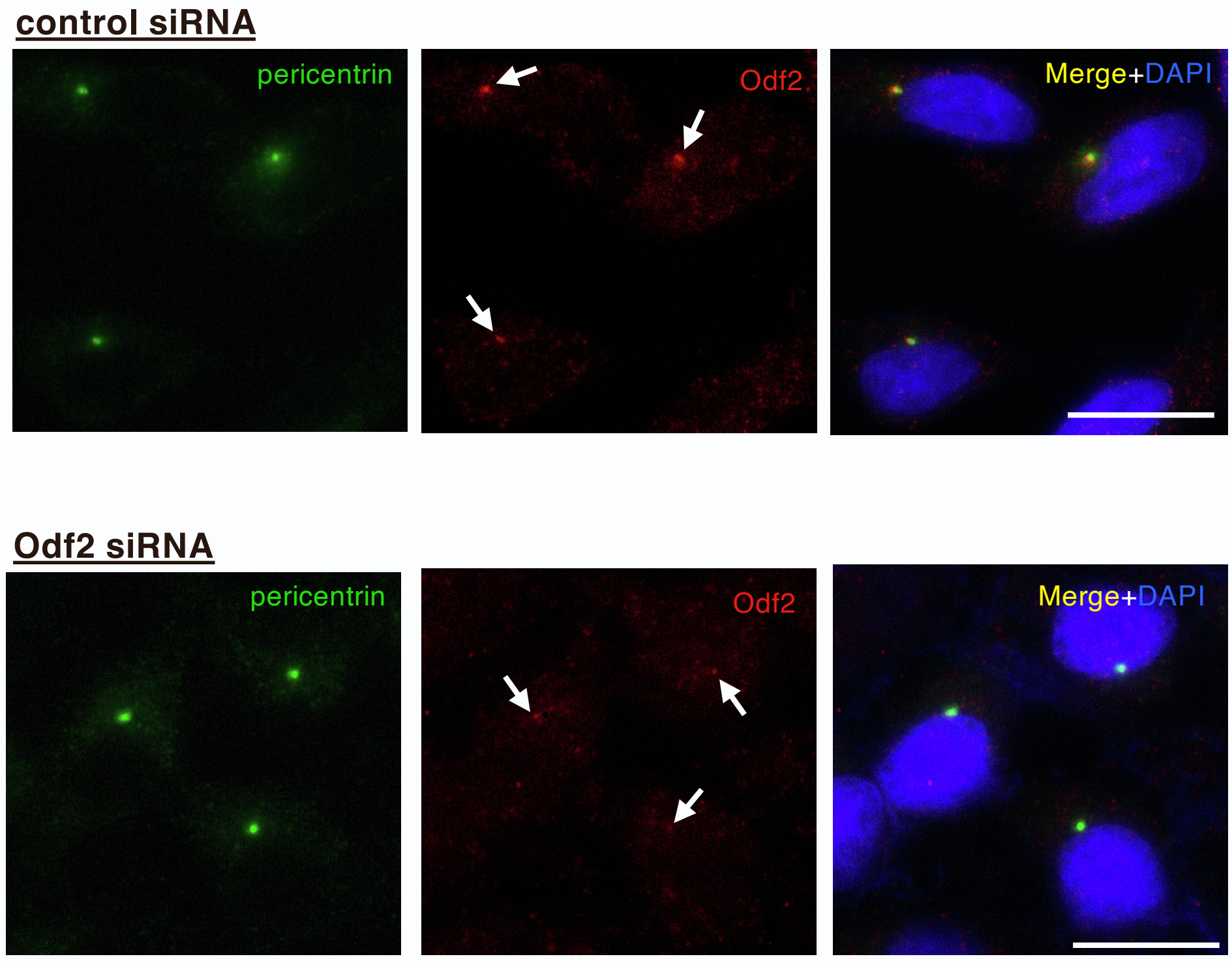
control siRNA
pericentrin
Odf2
Merge+DAPI
Odf2 siRNA
pericentrin
Odf2
Merge+DAPI

B

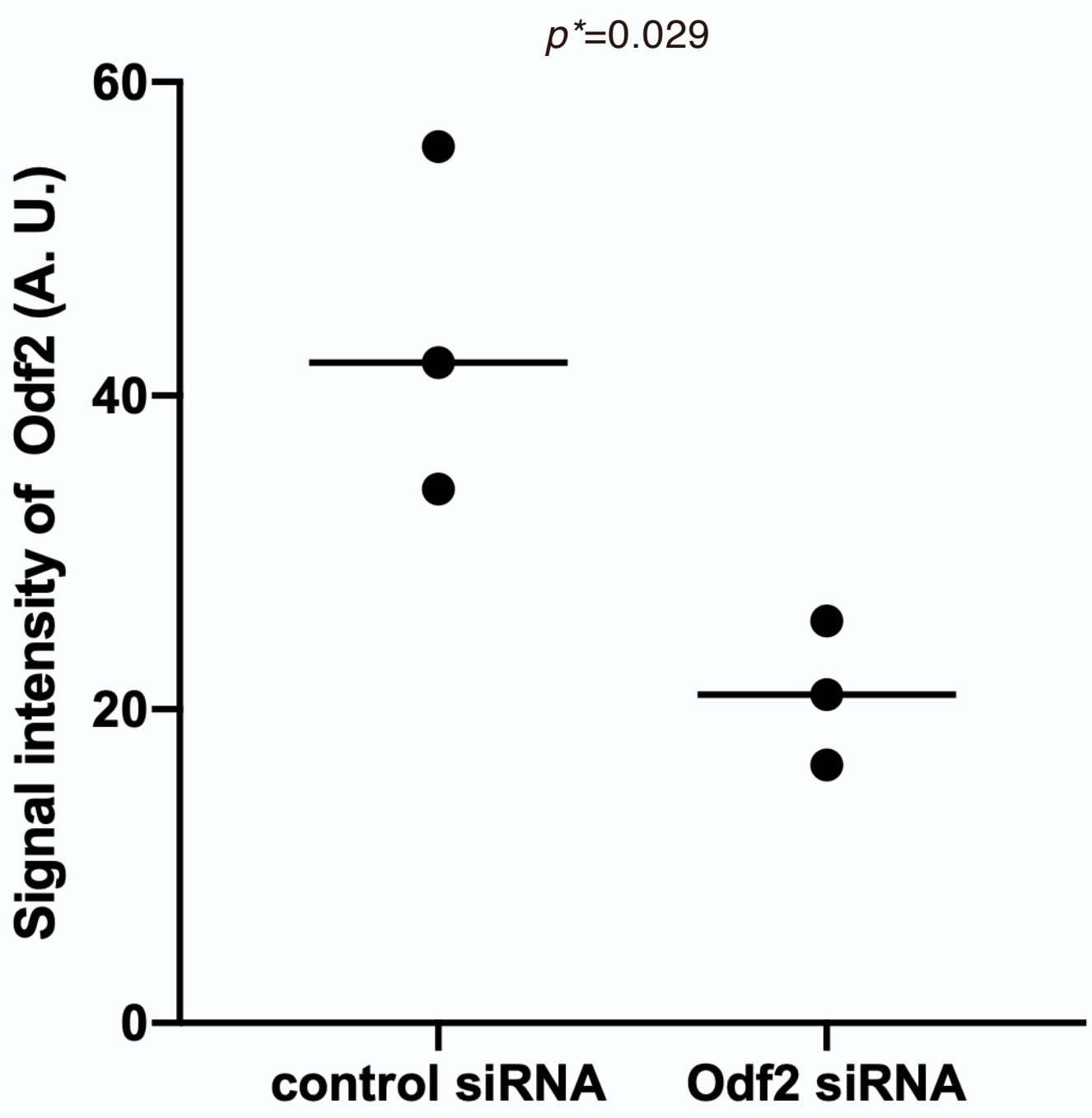
p*=0.029
60
40
20
0
Signal intensity of Odf2 (A. U.)
control siRNA
Odf2 siRNA

Figure S2

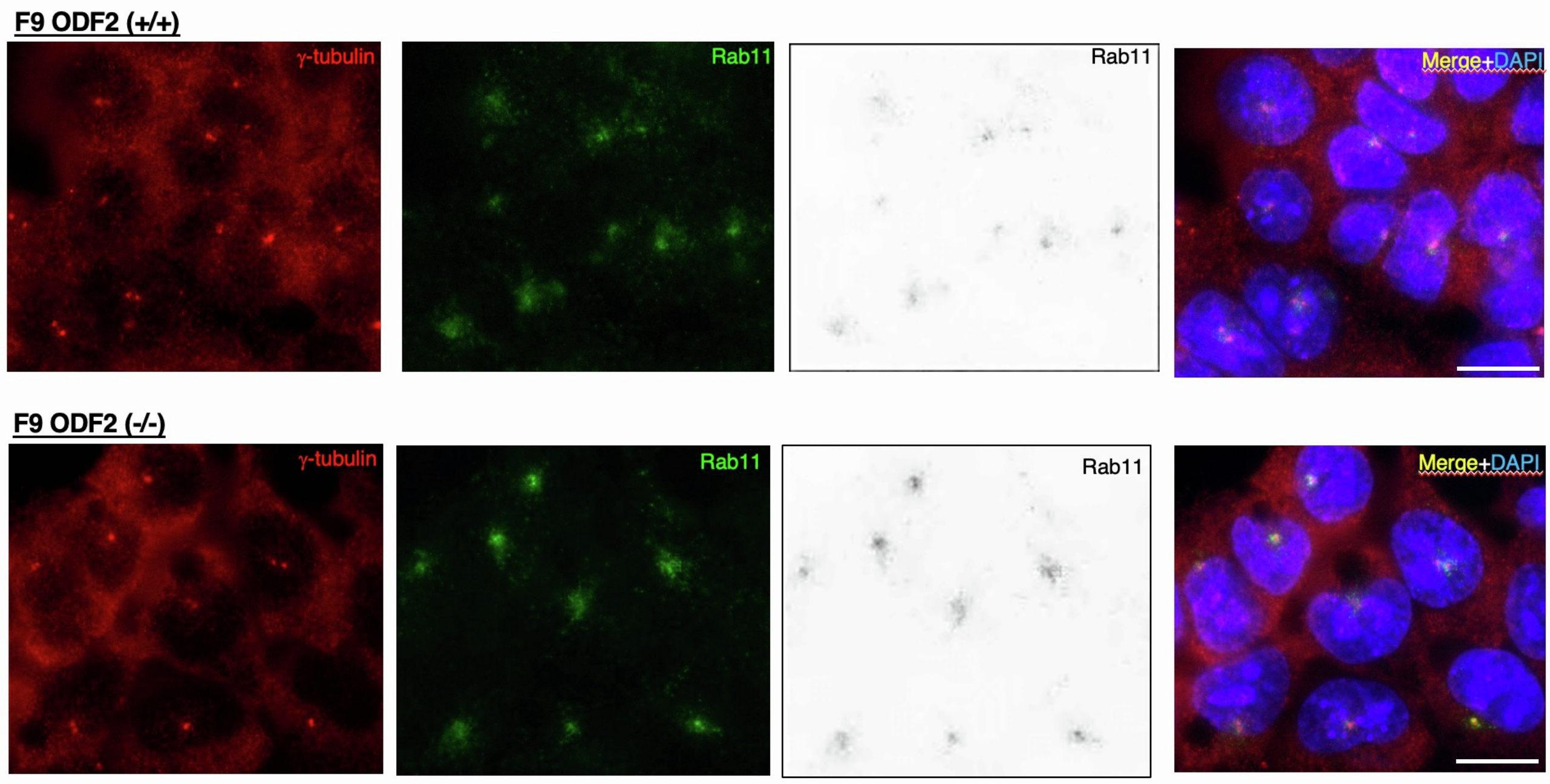
F9 ODF2 (+/+)
γ-tubulin
Rab11
Rab11
Merge+DAPI
F9 ODF2 (-/-)
γ-tubulin
Rab11
Rab11
Merge+DAPI

Figure S3

SK-N-DZ control siRNA (async)

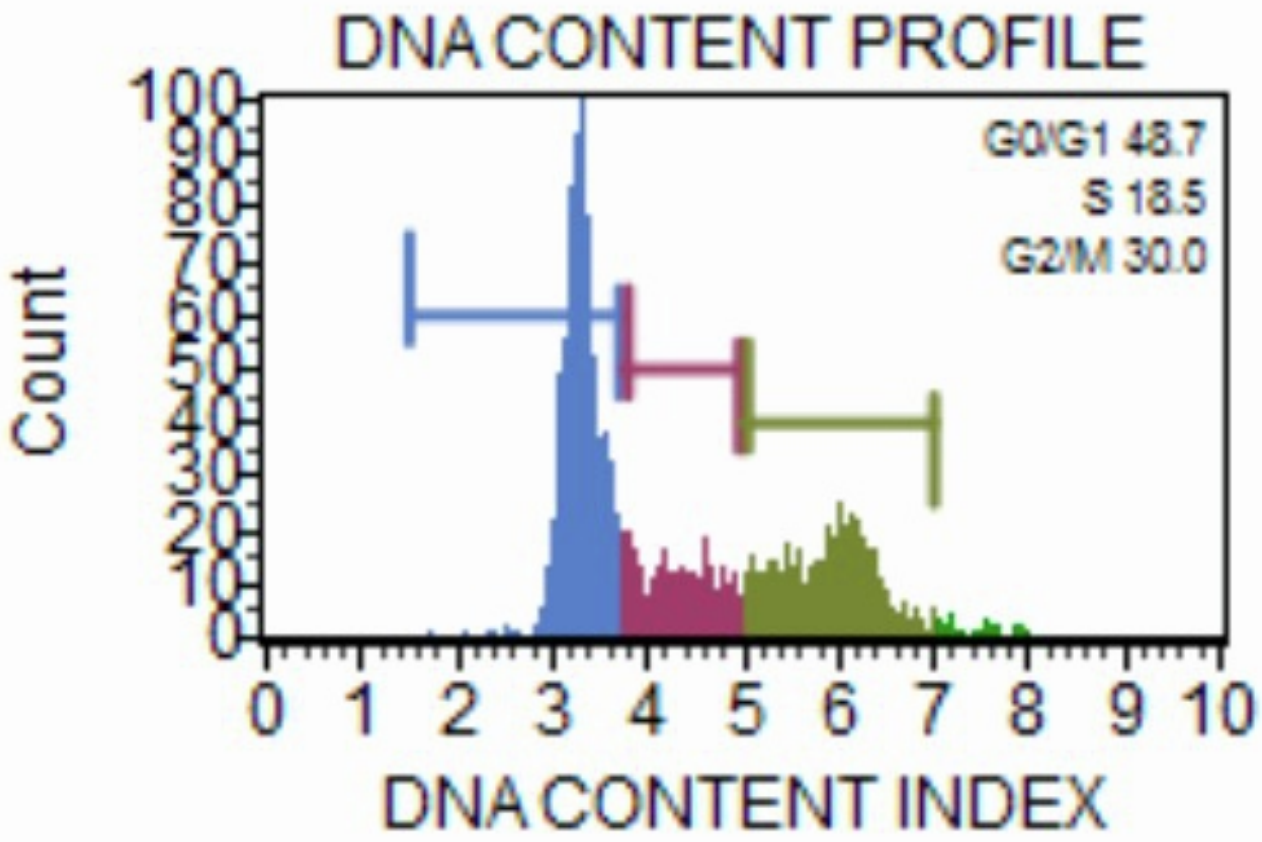

	G0/G1	S	G2/M
% Gated :	48.7	18.5	30.0
Mean :	3271.2	4333.5	5883.4
% CV :	7.7	8.4	8.2

SK-N-DZ control siRNA (serum (-) 48h)

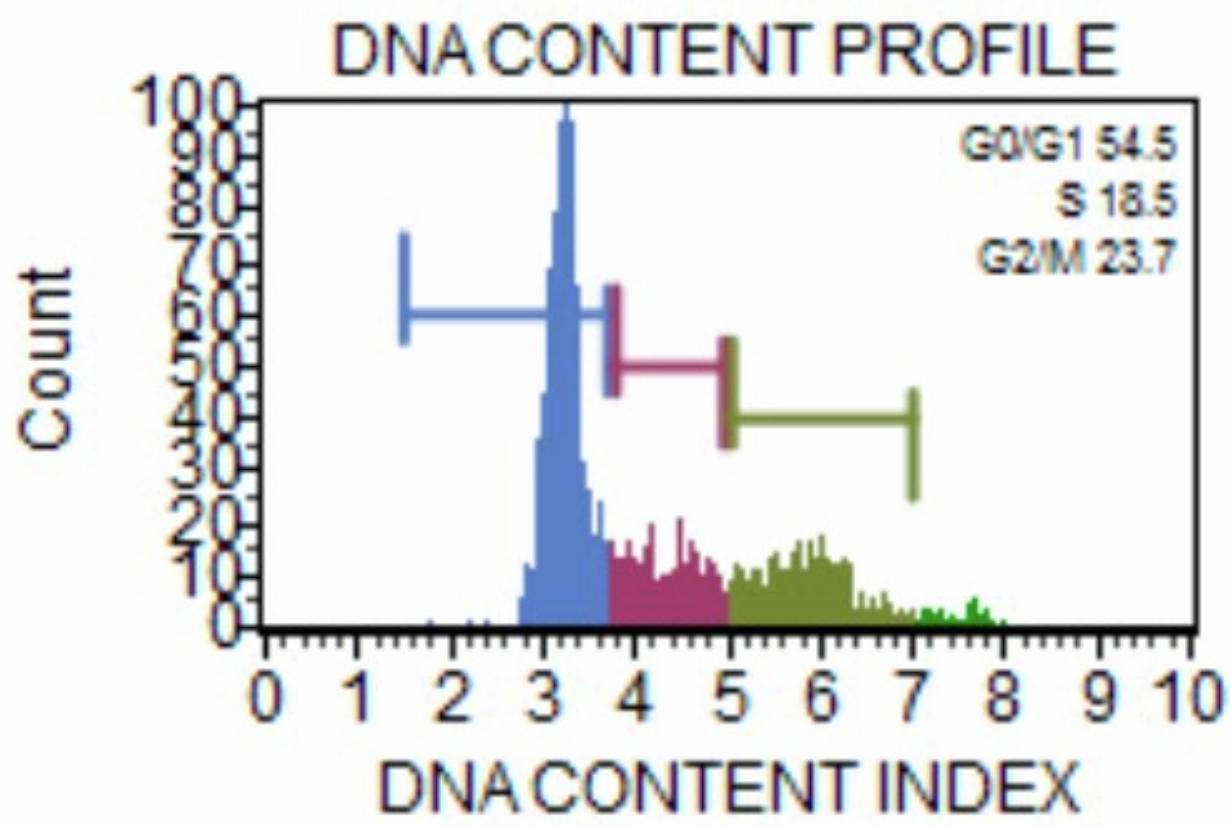

	G0/G1	S	G2/M
% Gated :	54.5	18.5	23.7
Mean :	3219.2	4335.9	5833.3
% CV :	7.5	8.4	8.3

SK-N-DZ CD133 siRNA (async)

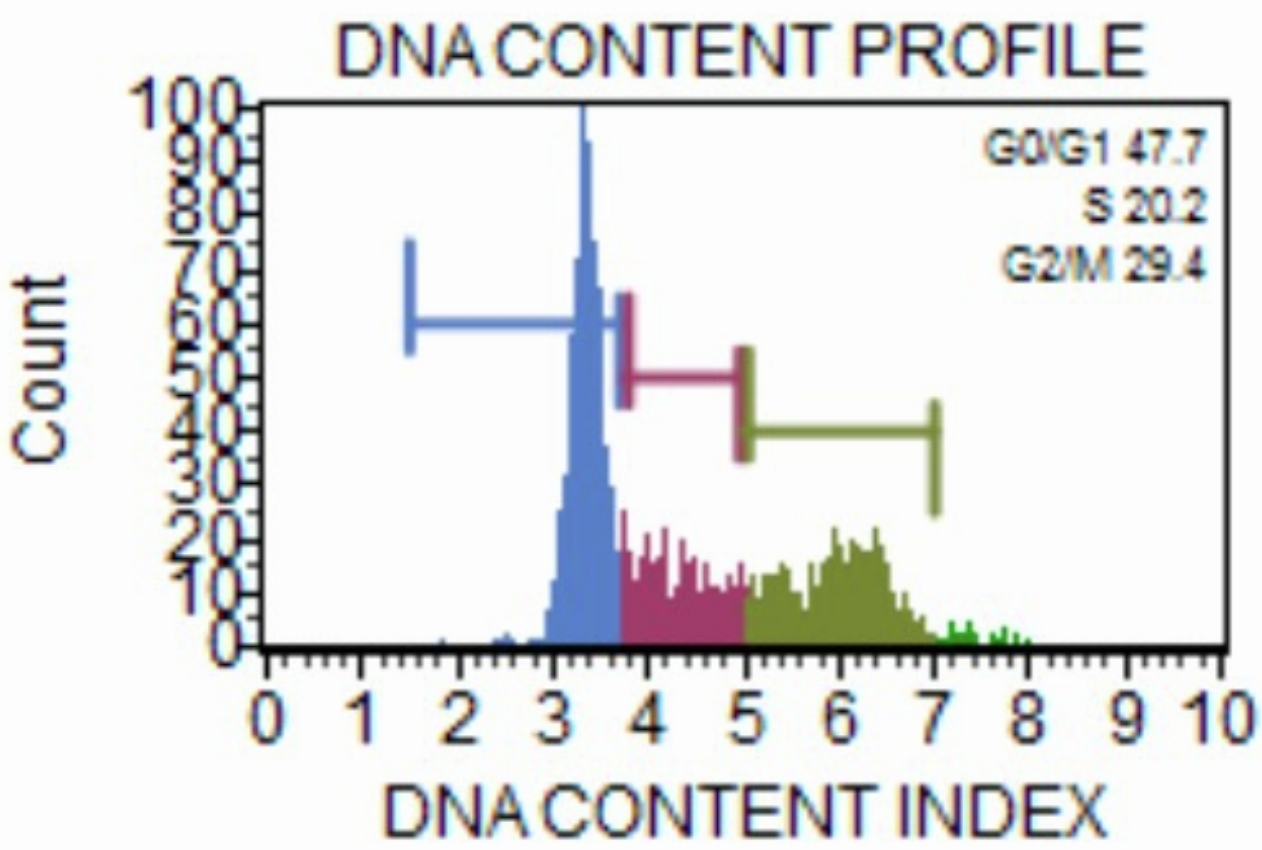

	G0/G1	S	G2/M
% Gated :	47.7	20.2	29.4
Mean :	3323.0	4332.8	5936.8
% CV :	7.2	8.5	8.1

SK-N-DZ CD133 siRNA (serum (-) 48h)

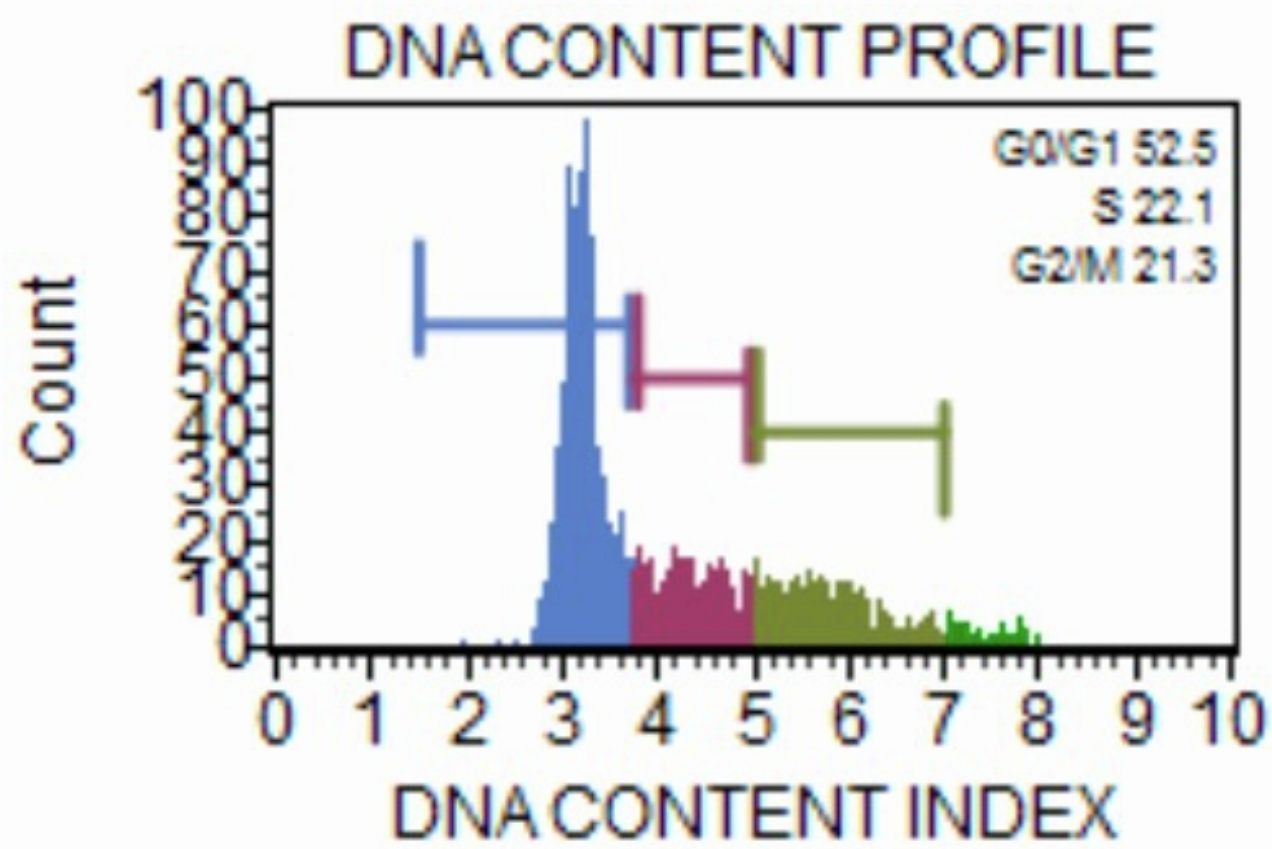

	G0/G1	S	G2/M
% Gated :	52.5	22.1	21.3
Mean :	3191.9	4347.6	5812.4
% CV :	7.7	8.5	8.9

Figure S4

Supplementary figure legends

Figure S1. Asymmetric co-inheritance of CD133- and Rab11-positive recycling endosomes during cytokinesis in SK-N-DZ cells, Related to Figure 3

(A) Representative immunostaining images of cytokinesis in SK-N-DZ cells. Pericentrin is green, CD133 is red, and DAPI (DNA) is blue. Arrows show centrosomes and arrowheads show the pericentrosomal localization of CD133. Phase contrast images are also shown. Scale bars, 10 µm. Black and white images show the cytokinesis pair analysis of SK-N-DZ using the image analysis software, Image J.

(B) Quantification of cells with the symmetric or asymmetric localization of CD133 in (A). **Data are presented as mean ± SEM from n = 3 independent experiments**; **45 cells were analyzed. Statistical significance was determined using a Student's t-test with equal variance ($p < 0.0001$).**

(C) Representative images of CD133 and Rab11 in SK-N-DZ cells. CD133 is red, Rab11 is green, and DAPI (DNA) is blue. Phase contrast images are also shown. Scale bars, 10 µm.

Figure S2. Knockdown of Odf2 in SK-N-DZ cells, Related to Figure 4

(A) Representative images of pericentrin and Odf2 in SK-N-DZ cells transfected with control or Odf2 siRNA. Pericentrin is green, Odf2 is red, and DAPI (DNA) is blue. Scale bars, 10 µm.

(B) The signal intensities of Odf2 in SK-N-DZ cells transfected with control (n=30) or Odf2 siRNA (n=30) are shown as dot graphs. **Data are presented as mean ± SEM from n = 3 independent experiments**; **30 cells per group were analyzed. Statistical**

significance was determined using a Student's t-test with equal variance ($p = 0.029$).

Figure S3. Rab11 localizes to centrosomes in F9 cells, Related to Figure 4

Representative images of γ-tubulin and Rab11 in F9 Odf2(+/+) and F9 Odf2(-/-) cells. γ-Tubulin is red, Rab11 is green, and DAPI (DNA) is blue. Scale bars, 10 μm. Black and white images show the signal intensities of Rab11.

Figure S4. Percentage of SK-N-DZ cells transfected with control or CD133 siRNA with or without 10% serum for 48 h in G0/G1, G2/M, and S phases, Related to Figure 5

The percentage was estimated by triplicate measurements using a FACS analysis (Muse Cell Analyzer) and the Muse cell-cycle analysis program.
